# Genome-Wide Identification and Expression Analysis of Senescence-Associated Genes in Grapevine (*Vitis vinifera* L.) Reveal Their Potential Functions in Leaf Senescence Order

**DOI:** 10.3390/ijms232112731

**Published:** 2022-10-22

**Authors:** You-Mei Li, Meng-Hao Sun, Xuan-Si Tang, Chao-Ping Wang, Zhao-Sen Xie

**Affiliations:** 1 College of Horticulture and Plant Protection, Yangzhou University, Yangzhou 225009, China; 2 Shandong Academy of Grape, Jinan 250000, China

**Keywords:** grapevine, senescence, leaf order, chlorophyll, ABA, gene expression

## Abstract

Natural leaf senescence is an acclimation strategy that enables plants to reallocate nutrients. In the present study, interestingly, we found that the basal mature leaves of grapevine primary shoots (P) exhibited the earliest senescence, followed by the apical young leaves of secondary shoots (ST), and then the basal mature leaves of secondary shoots (S). The Chl level decreased with the extent of leaf senescence. According to the genome-wide identification and expression analysis, sixteen senescence-associated genes (SAGs) involved in Chl breakdown were identified in the grapevine genome. Their expression patterns showed that the transcript changes in *VvSGR*, *VvPPH2*, and *VvFtsH6-2* corresponded to the changes in Chl content among P, S, and ST. The changes in the transcription of *VvNYC1*, *VvSGR*, *VvPAO1*, *VvPAO2*, *VvPAO4*, *VvPPH1*, *VvPPH3,* and *VvFtsH6-1* only contributed to low Chl levels in P. The cis-element analysis indicated that these SAGs possessed several light- and hormone-responsive elements in their promoters. Among them, ABA-responsive elements were found in twelve of the sixteen promoters of SAGs. Correspondingly, ABA-signaling components presented various changes in transcription among P, S, and ST. The transcription changes in *VvbZIP45* and *VvSnRK2.1* were similar to those in *VvSGR*, *VvPPH2*, and *VvFtsH6-2*. The other nine ABA-signaling components, which included *VvRCAR2*, *VvRCAR4*, *VvRCAR6*, *VvRCAR7*, *VvRCAR2*, *VvPP2C4*, *VvPP2C9*, *VvbZIP25*, and *VvSnRK2.3*, were highly expressed in P but there was no difference between S and ST, with similar expression patterns for *VvNYC*1, *VvSGR*, *VvPAO1*, *VvPAO2*, *VvPAO4*, *VvPPH1*, *VvPPH3*, and *VvFtsH6-1*. These results suggested that the senescence of P and ST could be regulated by different members of Chl breakdown-related SAGs and ABA-signaling components. These findings provide us with important candidate genes to further study the regulation mechanism of leaf senescence order in grapevine.

## 1. Introduction

Leaf senescence is a complex but orderly physiological process that enables plants to remobilize nutrients from old leaves to developing organs [1]. It is induced both by endogenous signals such as aging and by various exogenous factors such as stress [2]. However, a series of senescence syndromes occur irrespective of stimuli that cause leaf senescence, including programmed cell death and the ordered degradation of the macromolecules [3]. Among the other degradation processes, chlorophyll (Chl) catabolism into non-green derivatives leads to a visual manifestation of senescence syndromes, i.e., leaf yellowing via a multi-step catabolic pathway [4]. The Chl b-to-a conversion is the first step of Chl degradation; it is catalyzed sequentially by two Chl catabolic enzymes (CCEs), i.e., Chl b reductase (CBR) and droxymethyl Chl a reductase (HCAR) [5,6]. The former is encoded by two genes, *NON-YELLOW COLORING1* (*NYC1*) and *NYC1-LIKE* (*NOL*), in rice (*Oryza sativa*) [7] and Arabidopsis [5]. The next step is the removal of the Mg atom from Chl a, followed by the hydrolysis reactions. The *PPH* gene has been identified to encode pheophytinase, which catalyzes hydrolysis reactions in Arabidopsis [8]. The resulting pheophorbide a is oxygenolytically opened and further reduced to a primary nonfluorescent Chl catabolite by pheophorbide a oxygenase (PAO) and red Chl catabolite reductase (RCCR) [9]. Specifically, mutants deficient in *HCAR* (*hcar*) maintained green leaves during dark-induced and/or natural senescence, while *HCAR*-overexpressing plants exhibited more tolerance to reactive oxygen species than the wild type in rice (*Oryza sativa*) [10] and accelerated leaf yellowing in Arabidopsis [11]. The *pph*-1 mutant showed a stay-green phenotype since it was unable to degrade chlorophyll during senescence in Arabidopsis [12]. *LpNOL* RNA interference (*NOLi*) in perennial ryegrass (*Lolium perenne* L.) has been shown to significantly block Chl degradation in senescent leaves and also delayed the initiation and progression of leaf senescence [13]. In addition to CCEs, chloroplast protein NYE1, also known as STAY-GREEN1 (SGR1) has been shown to interact with CCEs at light-harvesting complex II (LHC II) for chlorophyll detoxification during leaf senescence in Arabidopsis [14,15]. The Arabidopsis genome processes three *SGR* homologs, i.e., *SGR1/NYE1*, *SGR2*, and *SGRL*. *SGR1/NYE1* has been characterized to promote Chl breakdown during leaf senescence in many plants [14], while *SGRL* contributes to Chl degradation in pre-senescent leaves [16]. By contrast, *SGR2* negatively regulates the degradation of Chl under age-, dark-, and stress-induced senescence conditions [17]. FtsHs are a family of ATP-dependent metalloproteases [18]. The chloroplast-located protease FtsH6 has been identified as being responsible for the degradation of LHC II during high-light acclimation and dark-induced senescence by using a reversed genetic approach [19]. These genes, such as *CCEs*, chloroplast protein, and *FtsHs* protease, are thought to be a type of senescence-induced senescence executers (SIEs), whose high expression levels directly result in senescence syndromes [20]. Recently, a study confirmed three abscisic acid (ABA)-responsive element (ABRE)-binding transcription factors (ABF2, ABF3, and ABF4) that functioned as the direct positive regulators of two CCEs (*PAO* and *NYC1*) to promote ABA-mediated Chl breakdown and leaf senescence [21]. Zhao et al. (2016) [22] found that *PYL9* over-expressing plants exhibited dramatically increased drought-induced leaf senescence and ABA-promoted leaf senescence via activating core ABA-signaling pathways (PP2Cs-SnRKs-ABFs). These genes belong to the senescence-induced senescence regulators (SIRs) group, in the context that they are activated during leaf senescence and regulate leaf senescence [20].

Grapevine, a worldwide important crop with highly commercial products such as table berries, juice, and wines, presents a full seasonal senescent syndrome. Leaf senescence is tightly related to crop yield and biomass production and needs massive changes in the expression level of SAGs [23]. A number of SAGs, including SIEs and SIRs, have been characterized in model plants [11,17,21]. Most recently, the A NAC transcription factor (DRL1) has been reported to be involved in the leaf senescence of grapevine [24]. *CitPPH* was directly regulated by an ethylene response factor involved in chlorophyll degradation during citrus fruit ripening [25]. However, genome-wide studies on a number of SAGs have still not been conducted in fruit trees, such as grapevine. During our experiment, we found that there was a certain order of leaf senescence in grapevine during autumn. The order of leaf yellowing depended on the leaf position. The aim of this study was to further investigate leaf senescence order in grape shoots, focusing on the changes in Chl contents and the genomic identification and expression analysis of Chl-related SAGs. The expression profiles of these genes in different nodes of leaves were indicative of distinct sets of Chl-related SAGs controlling the order of leaf yellowing. In addition, evidence was provided that ABA-related SAGs were also involved in the regulation of the senescence order of grapevine leaves.

## 2. Results

### 2.1. The Impact of Leaf Position on Senescence of Grapevine Leaves

**Figure 1:**
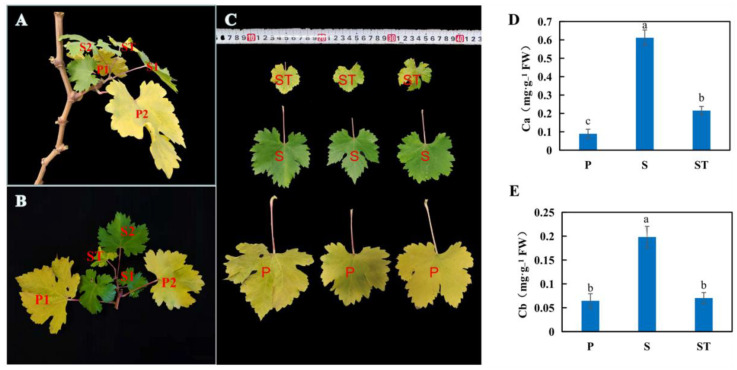
Leaf senescence depends on the leaf’s position in the grapevine. (**A**–**C**) Photographs of grapevine leaves with different extents of leaf senescence; (**D**,**E**) the content of chlorophyll a (Ca) and chlorophyll b (Cb). Values represent the means ± SD of 3 biological replicates. Different lowercase letters represent significant differences at *p*-value < 0.05.

Generally, leaf senescence in shoots occurs gradually from bottom to top. In the present study, the bottom leaves on primary shoots, indeed, turned yellow first. Interestingly, we observed senescence in the top leaves of secondary lateral shoots prior to the bottom leaves of secondary lateral shoots during natural autumn leaf senescence (Figure 1A,B). The leaves were collected and divided into three groups: the leaves on the first to second nodes of primary shoots (P group), the leaves on the first to second nodes of secondary shoots (S group), and the leaves on the tip of secondary shoots (ST group) (Figure 1C). Consistent with their senescence order, the chlorophyll a (Chl a) level in the S group was the highest, followed by the ST group, and the Chl a content in the P group was the lowest (Figure 1D). The Chl b content of the S group was obviously higher than that of the P and ST groups, while no difference was observed between the P and ST groups (Figure 1E).

### 2.2. Genome-Wide Identification and Characterization of Chl Degradation-Related SAGs in Grapevine Leaves

After using seven Chl degradation-related SAGs, which were functionally identified as transgenic Arabidopsis [26] in queries against the grapevine genome database, sixteen putative Chl degradation-related SAGs were identified in grapevine leaves (Table 1). The grapevine SAGs were placed into seven groups and named based on their locations in specific chromosomes: three *NOL* (*VvNOL1*, *2*, and *VvNYC1*), two *NYE1/SGR1* (*SGR* and *SGRL*), one *ACD2/RCCR* (*VvRCCR*), one *HCAR* (*VvHCAR*), four *PAO/ACD1* (*VvPAO 1*, *2*, *3*, and *4*), three PPH (*VvPPH 1*, *2*, and *3*), and two *FtsH6* (*VvFtsH6-1* and *VvFtsH6-2*). The exon-intron distributions of the grape SAGs presented variations among different groups (Figure S1). Twenty potentially conserved motifs were identified with *E*-values < 10^−5^, and SAGs within each class possessed similar motifs (Figure S2).

**Figure 2:**
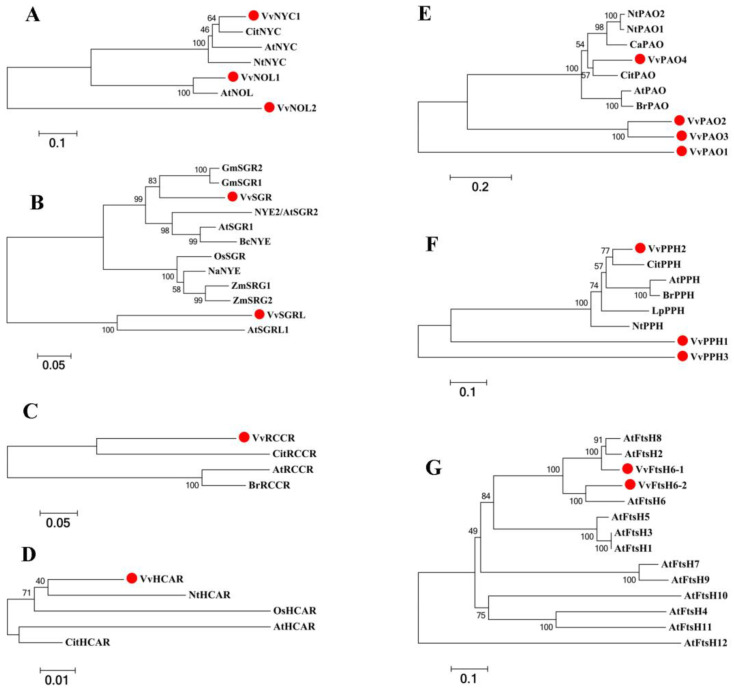
Phylogenetic analysis of Chl degradation-related SAGs. (**A**) Phylogenetic analysis of NYC/NOL from grapevine, Arabidopsis (*Arabidopsis thaliana*), tobacco (*Nicotiana tabacum*), and sweet orange (*Citrus sinensis*). (**B**) Phylogenetic analysis of SGR/SGRL from Arabidopsis (*Arabidopsis thaliana*), cabbage (*Brassica campestris*), soybean (*Glycine max*), rice (*Oryza sativa*), maize (*Zea mays*), and bamboo (*Neosinocalamus affinis*). (**C**) Phylogenetic analysis of RCCR from grapevine, Arabidopsis (*Arabidopsis thaliana*), cabbage (*Brassica rapa* var. parachinensis), and sweet orange (*Citrus sinensis*). (**D**) Phylogenetic analysis of HCAR from grapevine, Arabidopsis (*Arabidopsis thaliana*), sweet orange (*Citrus sinensis*), tobacco (*Nicotiana tabacum*), and rice (*Oryza sativa*). (**E**) Phylogenetic analysis of PAO from grapevine, Arabidopsis (*Arabidopsis thaliana*), sweet orange (*Citrus sinensis*), tobacco (*Nicotiana tabacum*), cabbage (*Brassica rapa* var. parachinensis), and pepper (*Capsicum annuum*). (**F**) Phylogenetic analysis of PPH from grapevine, Arabidopsis (*Arabidopsis thaliana*), tobacco (*Nicotiana tabacum*), sweet orange (*Citrus sinensis*), cabbage (*Brassica rapa* var. Parachinensis), and perennial ryegrass (*Lolium perenne*). (**G**) Phylogenetic analysis of FtsH from grapevine and Arabidopsis (*Arabidopsis thaliana*).

The phylogenetic tree (Figure 2A) showed that the *NOL* genes in grapevine, Arabidopsis (*Arabidopsis thaliana*), tobacco (*Nicotiana tabacum*), and sweet orange (*Citrus sinensis*) were resolved into three distinct groups. *VvNYC* and other *NYC* orthologs in Arabidopsis (*Arabidopsis thaliana*), tobacco (*Nicotiana tabacum*), and sweet orange (*Citrus sinensis*) clustered into one subgroup, *VvNOL1* clustered together with *AtNOL*, while the only member comprising the third subgroup was *VvNOL2*. *SGR* orthologs of grapevine, Arabidopsis (*Arabidopsis thaliana*), and cabbage (*Brassica campestris*) were clustered in a subgroup. *VvSGRL* was clustered with *AtSGRL* (Figure 2B). The *RCCR* orthologs were divided into two subclusters; *VvRCCR* and *CitRCC*R were clustered in one subcluster (Figure 2C). *VvHCAR* clustered closely with *NtHCAR* (Figure 2D). *VvPAO4* was clustered into a subgroup with characterized *PAO* orthologs of tobacco (*Nicotiana tabacum*), pepper (*Capsicum annuum*), sweet orange (*Citrus sinensis*), cabbage (*Brassica rapa var. parachinensis)*, and Arabidopsis (*Arabidopsis thaliana*) (Figure 2E). *VvPPH4* was divided into a subgroup with characterized *PPH* orthologs of tobacco (*Nicotiana tabacum*), sweet orange (*Citrus sinensis*), cabbage (*Brassica rapa* var. Parachinensis), perennial ryegrass (*Lolium perenne*), and Arabidopsis (*Arabidopsis thaliana*), while *VvPPH1* and *VvPPH3* were divided into two subclusters (Figure 2F). *VvFtsH6-1* and *VvFtsH6-2* clustered into subgroups with *AtFtsH6*, *AtFtsH2*, and *AtFtsH8* (Figure 2G).

### 2.3. Cis-Acting Regulatory Element Analysis of SAG Promoters

The cis-acting regulatory elements of the SAG promoters were analyzed (Table 2). Except for a common cis-acting element in promoter and enhancer regions, different numbers and types of light-response elements were found in promoters of most SAG members, such as ACE, AE-box, G-box, TCT-element, Box4, GATA-motif, and MRE. The hormone-responsive elements were also identified in the SAG members. ABRE, the cis-acting element involved in abscisic acid responsiveness, was found in promoters of *NYC1*, *NOL2*, *SGRL*, *SGR*, *RCCR*, *HCAR*, *PAO1*, *PAO2*, *PAO4*, *PPH2*, *FTsH6-1*, and *FTsH6-2*. One auxin-responsive element (AuxRR-core) was observed in promoters of *PAO3* and *PAO4*; two MeJA-responsive elements (CGTCA-motif and TGACG-motif) presented in promoters of *NOL2*, *SGRL*, *PAO3*, *PAO4*, and *PPH2*. Gibberellin-responsive elements, i.e., P-box and GARE-motif, were found in different promoters of SAGs. The former was in the promoters of *HCAR*, *PAO2*, *PPH2*, *PPH3*, *FTsH6-1*, and *FTsH6-2*; the latter was in the promoters of *NOL2*, *SGR*, *PAO1*, *PAO2*, *PPH2*, *PPH3*, *FTsH6-*1, and *FTsH6-2*. TCA-element, a cis-acting element involved in salicylic acid responsiveness, was identified in the promoters of *NOL2*, *SGRL*, *SGR*, *RCCR*, *HCA*R, *PAO3*, *PPH1*, *PPH2*, *PPH3*, and *FTsH6-1*.

### 2.4. Chl Degradation-Related SAGs Are Characterized by Diverse Expression Profiles in Different Leaf Positions of Grape Shoots

**Figure 3:**
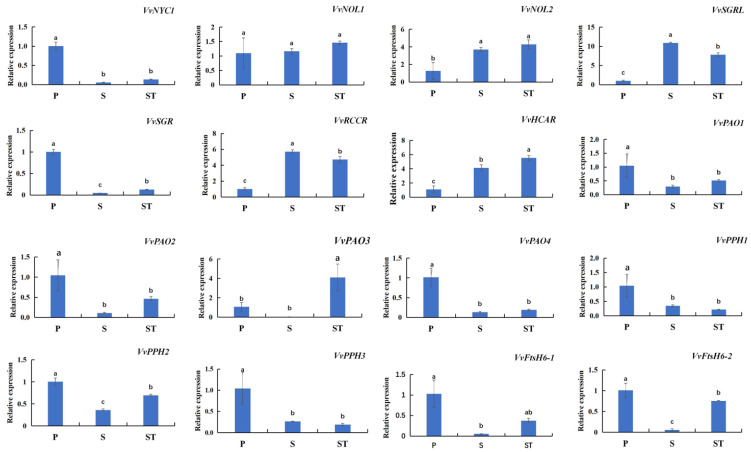
The expression patterns of Chl degradation-related SAGs in grapevine leaves with different extents of leaf senescence. Values represent the means ± SD of three biological replicates. Different lowercase letters represent significant differences at *p*-value < 0.05.

To identify the causal relationships between the order of grapevine leaf senescence and the transcript abundance of SAGs involved in Chl degradation, the expression level of 16 Chl degradation-related SAGs were quantified in different leaf nodes on grape shoots (Figure 3). In total, 15 SAGs were found to be differently expressed at one or three leaf positions: *VvSGRL* and *VvRCCR* were characterized by high expression levels in basal leaves on the secondary shoots (S) and low expression levels in basal leaves on primary shoots (P); *VvSGR*, *VvPPH*, *VvFtsH6-1*, and *VvFtsH6-2* presented the highest expression level in P, but the lowest expression level in S; and *VvHCAR* showed increasing expression from P to ST. The expression level of the other six SAGs, including *VvNYC1*, *VvPAO1*, *VvPAO2*, *VvPAO4*, *VvPPH1*, and *VvPPH1*, were obviously higher in P as compared with S and ST, while *VvNOL2* presented the opposite expression pattern. *VvPAO3* exhibited a significantly higher expression level in ST but no differences between P and S. The diverse expression profiles of these genes in different leaf positions could suggest the specific function of each gene during leaf senescence.

### 2.5. ABA-Related SAGs Are Characterized by Consistent Expression Profiles with Chl Degradation-Related SAGs in Different Leaf Positions of Grape Shoots

**Figure 4:**
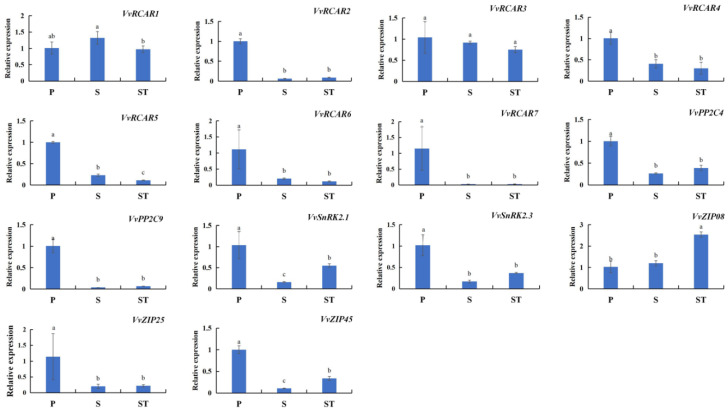
The expression patterns of ABA-signaling components in grapevine leaves with different extents of leaf senescence. Values represent the means ± SD of 3 biological replicates. Different lowercase letters represent significant differences at *p*-value < 0.05.

ABA is a well-known regulator of plant senescence. Twelve out of sixteen Chl degradation-related SAGs were found to contain ABA-responsive elements in their promoters. Therefore, 14 orthologs of ABA-signaling components were focused on the transcription changes in the different leaf positions of shoots (Figure 4). Five out of seven ABA receptors presented obviously high expression levels in P, but low levels in S and ST, including *VvRCAR2*, *VvRCAR4*, *VvRCAR5*, *VvRCAR6*, and *VvRCAR7*. The ABA-signaling components, such as *VvPP2C4*, *VvPP2C9*, *VvSnRK2.3*, and *VvZIP25*, showed similar expression patterns. *VvRCAR3* exhibited no significant differences among the three leaf positions, while *VvRCAR1* was most abundant in S. *VvSnRK2.3. VvZIP25* was significantly differentially expressed among the three leaf positions and presented the highest abundance in P but the lowest abundance in S. *VvZIP08* showed a high expression level in ST but low expression levels in S and P.

## 3. Discussion

Research on age-dependent leaf senescence has been mostly focused on the canopy or season level. However, there are few relevant studies on the senescence order of individual leaves. In this study, we found a type of branch group in which the leaves presented a certain senescence order. Specifically, leaf senescence was first exhibited on the mature basal leaves of primary shoots, followed by the top leaves of the secondary shoots, and then the basal leaves of the secondary shoots (Figure 1A,B). Therefore, we used the leaves of these shoots as experimental materials to explore the potential genes involved in the leaf senescence order of grapevine to therefore provide a theoretical basis for further research on the regulation mechanism of leaf senescence order in grapevine. The loss of chlorophyll is a significant hallmark of leaf senescence. Several findings have suggested that the Chl cycle activities are maintained at a low level unless the Chl b-to-a conversion is maintained under specific conditions or at specific developmental stages [27]. Indeed, high levels of Chl a and Chl b occurred in mature leaves (S) but sharply decreased in senescent leaves (P and ST) (Figure 1D). Chl metabolism pathways have been well studied; a number of genes related to Chl degradation have been identified and characterized as senescence-associated genes in several plant species, such as Arabidopsis [27], rice [10], and perennial ryegrass [13], but not previously reported in perennial fruit species. The Arabidopsis genome has identified two *NYC1* homologs (*NYC1* and *NOL*) [27], one *HCAR* [11], one *PPH* [12], one *PAO/ACD1* [28], one *RCCR/ACD2* [29], three *SGR* homologs (*SGR1/NYE1*, *SGR2*, and *SGRL*) [14], and one ATP-dependent metalloprotease number (AtFtsH6) [19] encoding *CBR*, *HCAR*, *PPH*, *PAO*, *RCCR*, *SGR,* and *AtFtsH6*, respectively, and playing roles as senescence executers during leaf senescence. In the present study, twelve genes encoding CCEs (one *NYC*, two *NOLs*, one *HCAR*, three *PPH*, four *PAO*, and one *RCCR*), two encoding chloroplast-located proteases (*VvFtsH6-1* and *VvFtsH6-2*), and two encoding chloroplast proteins (*SGR* and *SGRL*) were identified in the grapevine genome by using the above Arabidopsis orthologs as queries (Table 1). Significantly, of three *SGR1/SGRL* homologs (*SGR1/NYE1*, *SGR2*, and *SGRL*) present in the Arabidopsis genome, *SGR1/NYE1* [14] and *SGRL* [16] contribute to Chl breakdown in senescent leaves and pre-senescent leaves, respectively, while *SGR2* [17] negatively regulates Chl degradation under different senescence conditions. In this study, three candidate SGR1/SGRL sequences were obtained from the BLAST process; however, one of them was deleted because of its short length. Therefore, only two potential SGR1/SGRL sequences were obtained (Table 1). The phylogenetic tree analysis showed that the sequence named VvSGR clustered with AtSGR1, and VvSGRL clustered with AtSGRL (Figure 2). In addition, the expression level of *VvSGR* increased with leaf senescence, while *VvSGRL* showed high abundance in pre-senescent leaves (S) but low abundance in senescent leaves (P). Therefore, *VvSGR* could be the ortholog of Arabidopsis *SGR1*, and *VvSGRL* could be the *AtSGRL* ortholog. Further tests of gene expressions and Chl changes at different nodes of leaves showed that Chl content decreased with the extent of leaf senescence (Figure 1), and sixteen SAGs were characterized by distinct expression profiles at different nodes of leaves (Figure 3). The expression levels of ten genes, i.e., *VvNYC1*, *VvSGR*, *VvPAO1*, *VvPAO2*, *VvPAO4*, *VvPPH1*, *VvPPH2*, *VvPPH3*, *VvFtsH6-1*, and *VvFtsH6-2* were significantly high in P but low in S (Figure 3), which corresponded to low levels of Chl in P and high levels of Chl in S, respectively (Figure 1). However, only four genes (*VvSGR*, *VvPAO3*, *VvPPH2*, and *VvFtsH6-2*) had significantly different expressions between S and ST; their expression patterns corresponded to a low level of Chl in ST and a high level of Chl in S. This could suggest a complex regulation of cellular Chl degradation during leaf senescence. Among the differently expressed CCEs, the *PPH* expression level has been found to positively correlate with the extent of leaf senescence during the natural leaf maturation process [30]. Two *CBR* isoforms, *NYC1* and *NOL*, have been suggested to have a similar function in Chl metabolism in Arabidopsis but act at different developmental stages, resulting from their different mutant phenotypes and expression patterns [5,11]. Similarly, *VvNYC* expression patterns differed considerably from *VvNOL1* and *VvNOL2* in different nodes of grapevine leaves (Figure 3). A significant difference in *VvNYC1* expression was observed with the extent of leaf senescence, corresponding to the function that *NYC1* was involved with in naturally induced senescence in Arabidopsis [5,11]. Similar to rice and Arabidopsis [6,10], a single *HCAR* homolog was identified in the grapevine genome database (Table 1). Its expression pattern was similar to *VvNOL2* (Figure 3), which was consistent with the results reported for Arabidopsis [11]. Tanaka et al. (2003) [28] found three possible PAO-encoding genes in the Arabidopsis genome (*Tic55*, At2g24820; *ACD1*, At3g44880; and *ACD1-like*, At4g25650) and confirmed that only the *ACD1* gene encodes PAO. Using the ACD1 protein as the query sequence to blast the grapevine genome database, four VvPAO homologs were searched in the grapevine genome database. Their expression patterns indicated that all of them were involved in Chl breakdown at different nodes of leaves. Red Chl catabolite reductase (RCCR) catalyzes the conversion of red Chl catabolite (RCC) to red Chl catabolite (RCC), which is essential in Chl breakdown [27]. However, the expression changes of *VvRCCR* in different-aged leaves seem not to be related to Chl breakdown (Figure 3). Its function needs to be further characterized.

To explore potential SIRs regulating these SIEs, an analysis of the cis-element components of sixteen SIEs was performed. The results showed that some SIE members shared conserved core nucleotide sequences potentially recognized by abscisic acid (ABRE), auxin (AuxRR-core), methyl jasmonate (CGTCA-motif/TGACG-motif), gibberellin (TATC-box/GARE-motif/P-box), and salicylic acid (TCA-element) signaling (Table 2). Among them, ABRE is a downstream core nucleotide sequence recognized by ABRE-binding proteins (ABFs/AREBs) [31,32] and was identified in most (twelve out of sixteen) of the SIE promoters in the present study. A recent study reported that ABF4 activated the expression of *AtNYC1* and *AtPAO* by directly binding their promoters in mediating ABA-triggered Chl degradation and leaf senescence [21]. A number of cis-elements involved in light responsiveness were also found in the SIE promoters in addition to hormone-responsive elements, including ACE, AE-box, G-box, Box4, GATA-motif, MYE, and TCCC-motif. Many genes associated with Chl breakdown have been reported to respond to dark-induced senescence [5,11]. However, there are still limited reports on the binding specification for light-responsive transcription factors that may activate or deactivate SIE expression. The presence of these conserved cis-elements, including hormones and light, provides us with a strong clue for identifying potential transcription factors that may directly regulate Chl breakdown during the natural senescence of grapevine leaves, such as ABFs. In an attempt to uncover potential ABA-related SIRs involved in leaf senescence order, the expression patterns of seven ABA receptors identified in the grapevine genome (*VvRCAR1–7*), two of the major binding partners of the ABA receptor (*VvPP2C4* and *VvPP2C9*) [33], three *ABF3/4* orthologs (*VvbZIP08*, *VvbZIP25*, and *VvbZIP45*) [34], and two *VvSnRKs* expressed highly in leaves (*VvSnRK2.1* and *VvSnRK2.3)* [35] were investigated in leaves with different extents of leaf senescence. Similar to previous findings, ABA-signaling components are required for ABA-triggered Chl breakdown and leaf senescence [21]. In this study, ABA-signaling components showed differential expression patterns with the extent of Chl degradation and leaf senescence (Figure 4). It was observed that the transcription levels of *VvRCAR2*, *4*, *6*, 7, *VvPP2C4*, *9*, *VvbZIP25*, and *VvSnRK2*.3 were obviously high in P but showed no difference between S and ST, which presented similar expression patterns with six Chl degradation-related SAGs. The *ZIP25* ortholog in Arabidopsis (*ABF4*) has been confirmed to mediate ABA-triggered Chl degradation and leaf senescence by directly activating the expression of *AtNYC1* and *AtPAO* [21]. The expression patterns of *VvbZIP45* and *VvSnRK2.1* were similar to those of *VvSGR*, *VvPPH2*, and *VvFtsH6-2*, and their expression patterns corresponded to changes in Chl content in leaves with different extents of leaf senescence. The *PPH* orthologs in perennial ryegrass (*LpPPH*) have been shown to be positively related to the extent of leaf senescence and could be a direct downstream target gene of transcription factors in the ABA-signaling pathways [30]. It seems that ABA-signaling components are related to the leaf senescence order of grapevine, but the various changes in the transcription of Chl degradation-related SAGs and ABA-signaling components suggest that different regulatory networks could occur in leaves with different extents of leaf senescence. The integral regulatory network of Chl breakdown in leaves with different extents of leaf senescence remains to be discovered.

## 4. Materials and Methods

### 4.1. Plant Material and Sampling

Two-year-old vines of V. vinifera cv. ‘Huangjinmi’ were grown in potting soil conditions in a greenhouse at Yangzhou University, Yangzhou, Jiangsu Province of China (119°26′ E, 32°24′ N). The vines were grafted on rootstock varieties ‘Beta’ (Vitis riparia × V. labrusca). Seven uniform trees were selected and 18 branches arising from the lateral buds on the primary shoot and developing into primary and secondary lateral shoots were marked and used for sampling. Six branches served as one biological replicate. Leaves at the same positions were pooled and frozen in liquid nitrogen and then stored at −80 °C.

### 4.2. Measurement of Chl Content

For each replicate, 0.1 g of frozen leaves was extracted, and Chl a/b content was determined using the method reported by Burnison et al. (2011) [36].

### 4.3. Identification of Chl Degradation-Related SAGs in Grapevine Leaves

Arabidopsis thaliana Chl degradation-related SAGs were downloaded from the leaf senescence database LSD3.0 [26] and used as queries to identify genes encoding homologous proteins in the grape genome [37] using the BLAST program in the Phytozome comparative platform [38] with a 10^−5^ *E*-value as the threshold. The obtained grape SAG proteins were then aligned using a conserved domain search tool (http://www.ncbi.nlm.nih.gov/Structure/cdd/wrpsb.cgi (accessed on 19 April 2020)). Genes lacking conserved domains or that were less than 200 bp were discarded.

### 4.4. Phylogenetic Analysis, Gene Structure Analysis, and Promoter Analysis

The protein sequence of functionally characterized Chl degradation genes in Arabidopsis (*Arabidopsis thaliana*), tobacco (*Nicotiana tabacum*), sweet orange (*Citrus sinensis*), cabbage (*Brassica rapa* var. parachinensis), cabbage (*Brassica campestris*), rice (*Oryza sativa*), perennial ryegrass (*Lolium perenne*), maize (*Zea mays*), bamboo (*Neosinocalamus affinis*), and soybean (*Glycine max*) were downloaded from the leaf senescence database LSD3.0 [26] and then used for phylogenetic analysis with the MEGA 6.06 software via the neighbor-joining method and the bootstrap test replicated 1000 times. Gene exon-intron structures were generated using the online Gene Structure Display Server (http://gsds.cbi.pku.edu.cn accessed on 1 May 2020) [39]. Putative motifs for all of the SAG genes were analyzed using the MEME ver.4.11.1 program (http://meme-suite.org/tools/meme accessed on 1 May 2020) [40]. The promoter sequences were analyzed using the PlantCare database with default parameters [41].

### 4.5. Expression Analysis by Quantitative Real-Time Polymerase Chain Reaction (RT-qPCR)

Total RNA was extracted from frozen leaves using a CTAB-based method [42]. First-strand cDNA was synthesized with a PrimeScriptTM RT reagent Kit with gDNA Eraser (Perfect Real Time) (TaKaRa, Dalian, China). The primer pairs of SAGs for RT-qPCR were designed using Primer 6.0 and are listed in Supplementary Table S1. The RT-qPCR protocol was performed based on the manufacturer’s instructions with the SYBR Premix ExTaqTM II kit (Takara, Beijing, China) using the CFX Connect Real-Time PCR Detection System (Bio-Rad). ACTIN from grapes was used as a reference gene. The relative expression was calculated using the 2^−ΔΔCt^ method [43].

### 4.6. Statistical Analysis

The Chl content and RT-qPCR results were subjected to a one-way analysis of variance and the means were compared using the Tukey test with the SPSS software 22.0. Figures were constructed using Excel 2010.

## 5. Conclusions

In this work, we found that the grapevine leaves on primary and secondary shoots aged in a certain order in autumn. The Chl level decreased with the extent of leaf senescence and sixteen Chl breakdown-related SAGs were identified in grapevine leaves. The greater degree of Chl degradation in the basal mature leaves of grapevine primary shoots than that in the basal mature leaves of secondary shoots was associated with higher transcript levels of *VvNYC1*, *VvSGR*, *VvPAO1*, *VvPAO2*, *VvPAO4*, *VvPPH1*, *VvPPH2*, *VvPPH3*, and *VvFtsH6-1* in the former leaves. The greater degree of Chl degradation in the apical young leaves of secondary shoots than that in the basal mature leaves of secondary shoots was related to the higher expression levels of *VvSGR*, *VvPAO*3, *VvPPH2*, and *VvFtsH6-2* in the former leaves. ABA-responsive elements were mostly identified in the promoters of these SAGs. Further analysis of the expression pattern of ABA-signaling components suggested that ABA signaling was involved in regulating the order of leaf senescence, but the regulation mechanism of ABA-mediated Chl degradation could be different in different nodes of leaves. Further studies are required to fully understand the mechanism of the different extents of leaf senescence in different leaf orders of grapevine leaves.

## Figures and Tables

**Table 1:** Characteristics of the Chl degradation-related SAGs identified in the grapevine genome database.

Gene Name	Gene ID	Location	Peptide (aa)
*VvNYC1*	VIT_11s0016g03890.2	chr11:3174337..3179203−	518
*VvNOL1*	VIT_01s0010g00590.1	chr1:15587022..15619072−	317
*VvNOL2*	VIT_12s0035g01780.1	chr12:21916237..21924762−	265
*VvSGR*	VIT_02s0025g04660.1	chr2:4225612..4232509+	228
*VvSGRL*	VIT_18s0001g01210.1	chr18:1811441..1814300−	252
*VvRCCR*	VIT_07s0031g00680.1	chr7:16847972..1685168+	323
*VvHCAR*	VIT_05s0051g00070.2	chr5:10300193..10315728−	458
*VvPAO1*	VIT_04s0008g07020.1	chr4:7106897..7110246−	545
*VvPAO2*	VIT_06s0004g00610.1	chr6:769345..772779−	524
*VvPAO3*	VIT_06s0004g00620.1	chr6:780506..783167−	464
*VvPAO4*	VIT_06s0061g00790.1	chr6:18321351..18327161−	540
*VvPPH1*	VIT_04s0023g02010.1	chr4:18547350..18550496+	368
*VvPPH2*	VIT_13s0158g00180.2	chr13:21072015..21076223−	525
*VvPPH3*	VIT_16s0022g01340.2	chr6:780506..783167−	464
*VvFtsH6-1*	VIT_12s0028g01600.1	chr12:2304815..2308584+	695
*VvFtsH6-2*	VIT_14s0108g00590.1	chr14:29333100..29335715+	677

**Table 2:** Cis-Acting regulatory element analysis of Chl degradation-related SAGs promoters.

Element	Function	*Cis*-Element on SAGs Promoters															
		*NYC1*	*NOL1*	*NOL2*	*SGRL*	*SGR*	*RCCR*	*HCAR*	*PAO1*	*PAO2*	*PAO3*	*PAO4*	*PPH1*	*PPH2*	*PPH3*	*FtsH6-1*	*FtsH6-2*
ABRE	Abscisic acid responsiveness	**+**		**+**	**+**	**+**	**+**	**+**	**+**	**+**		**+**		**+**		**+**	**+**
ACE	Light responsiveness					**+**			**+**								
AE-box	Light response					**+**		**+**		**+**		**+**			**+**		**+**
ARE	Anaerobic induction	**+**	**+**		**+**	**+**	**+**	**+**	**+**	**+**	**+**	**+**	**+**	**+**	**+**	**+**	**+**
CAAT-box	Common cis-acting element in promoter and enhancer regions			**+**	**+**	**+**	**+**	**+**	**+**		**+**	**+**		**+**	**+**	**+**	**+**
AuxRR-core	Auxin responsiveness										**+**	**+**					
CGTCA-motif	MeJA-responsiveness			**+**	**+**						**+**	**+**		**+**			
G-box	Light responsiveness	**+**			**+**	**+**		**+**	**+**	**+**		**+**		**+**		**+**	**+**
TATC-box	Gibberellin-responsiveness						**+**						**+**	**+**	**+**		
TC-rich repeats	Defense and stress responsiveness		**+**					**+**						**+**			
TCA-element	Salicylic acid responsiveness			**+**	**+**	**+**	**+**	**+**			**+**		**+**	**+**	**+**		**+**
TCT-motif	Part of a light-responsive element	**+**	**+**	**+**		**+**		**+**			**+**	**+**		**+**		**+**	
TGACG-motif	MeJA-responsiveness			**+**	**+**						**+**	**+**		**+**	**+**		
Box4	Light responsiveness	**+**	**+**	**+**	**+**	**+**	**+**		**+**	**+**	**+**	**+**	**+**	**+**	**+**	**+**	**+**
GARE-motif	Gibberellin-responsive element			**+**		**+**			**+**	**+**			**+**	**+**		**+**	
P-box	Gibberellin-responsive element							**+**		**+**				**+**	**+**	**+**	**+**
GATA-motif	Part of a light-responsive element	**+**				**+**								**+**			
MRE	MYB binding site involved in light responsiveness	**+**	**+**										**+**			**+**	
AT1-motif	Part of a light-responsive module						**+**					**+**		**+**			
ATCT-motif	Light responsiveness							**+**						**+**			
I-box	Light-responsive element			**+**	**+**												
chs-CMA2a	Light-responsive element	**+**				**+**					**+**						
ABRE3a		**+**			**+**	**+**	**+**		**+**			**+**		**+**			**+**
ABRE4		**+**			**+**	**+**	**+**		**+**			**+**		**+**			**+**
TCCC-motif	Light-responsive element			+	+			+		+	+						

## Data Availability

Not applicable.

## Supplementary Materials

The following are available online at https://www.mdpi.com/article/10.3390/ijms232112731/s1.
