# Supplementary material for: Genome-Wide Identification and Expression Analysis of Senescence-Associated Genes in Grapevine (Vitis vinifera L.) Reveal Their Potential Functions in Leaf Senescence Order
Source: Int J Mol Sci. 2022 Oct 22;23(21):12731. doi: 10.3390/ijms232112731 (PMC9656468; doi:10.3390/ijms232112731)
Supplement: Supplementary file 1 [file ijms-23-12731-s001.zip › Supplementary Figures.pdf]

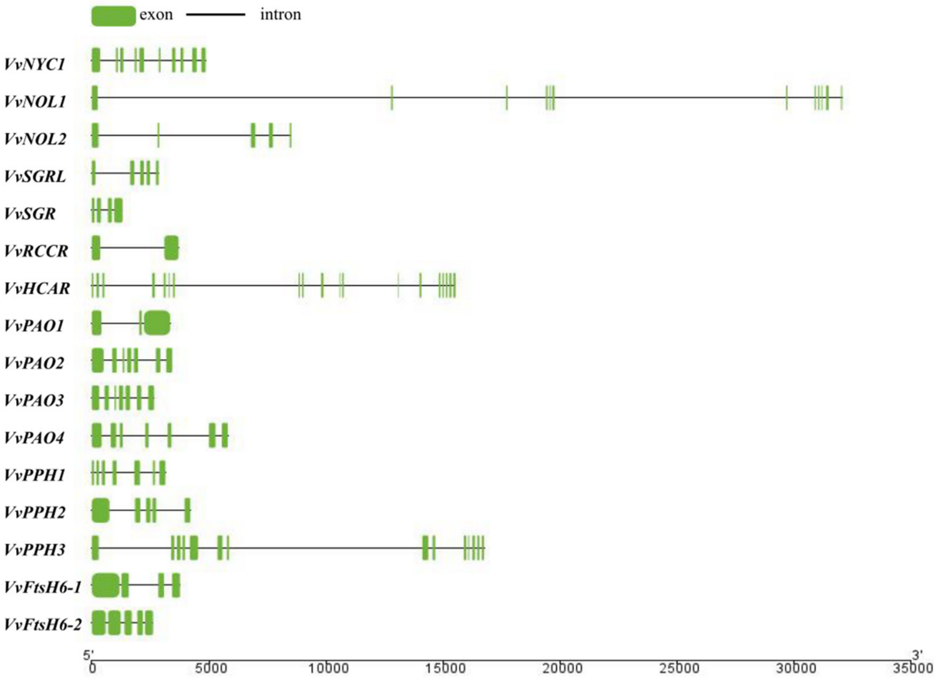

Supplementary Figure S1 The intron/exon organization of Chl degradation related SAGs.

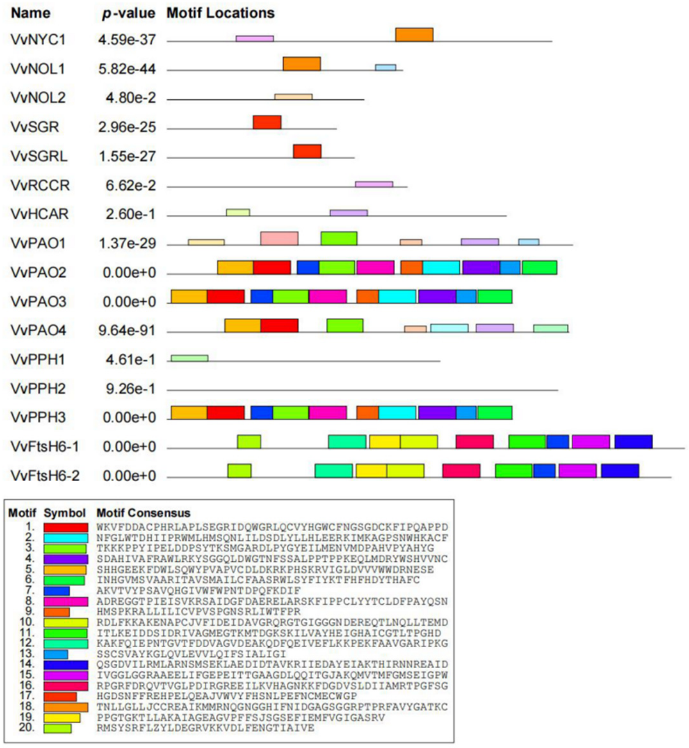

Supplementary Figure S2 Composition of conserved motifs of Chl degradation related SAGs.
