# Supplementary material for: Genome-Wide Identification and Expression Analysis of Senescence-Associated Genes in Grapevine (Vitis vinifera L.) Reveal Their Potential Functions in Leaf Senescence Order
Source: Int J Mol Sci. 2022 Oct 22;23(21):12731. doi: 10.3390/ijms232112731 (PMC9656468; doi:10.3390/ijms232112731)
Supplement: Supplementary file 1 [file ijms-23-12731-s001.zip › Supplementary Table S1.pdf]

Table S1. List of primers used for RT-qPCR.

Classification	Gene Name	Gene ID	Primer sequence(5′→3′)
Reference gene	*VvActin*	VIT_04s0044g00580.1	F:CTTGCATCCCTCAGCACCTT
			R:TCCTGTGGACAATGGATGGA
Chl degradation related SAGs	*VvNYC1*	VIT_211s0016g03890.2	F:GGTGAATTGGAGGAGCAAGAGAAG
			R:GGAAACCCTAACAAACACGGACTT
	VvNOL1	VIT_201s0010g00590.1	F:GGCGATTACTGCTTCCGTGTC
			R:AAGGTGAGGAAGGCGTGAGAG
	VvNOL2	VIT_212s0035g01780.1	F:GCTCTCATCAGTCGCCTCTTCT
			R:CGTGCCAACTCTTCAACACTACT
	VvSGRL	VIT_218s0001g01210.1	F:ACCCTCTCCCACTGTGACTTTAC
			R:ACTTCCTTCCACTCAGCAACCA
	VvSGR	VIT_202s0025g04660.1	F:ACGCTTACGCATAGTGACATAACA
			R:ATTCTGCCACCACCTCATCTCTT
	VvRCCR	VIT_207s0031g00680.1	F:CCTACATCTCTGGTCCTCATTCT
			R:AGTTGTCTGCGACTCTCCAAT
	VvHCAR	VIT_205s0051g00070.2	F:AGTGGACAGGGATAGTGACAACAA
			R:TCATCTGGCGTCCTGGCTAAG
	VvPAO1	VIT_204s0008g07020.1	F:GCGGAGTATGATTGGAAGGAAGAG
			R:CCTTGCCATCACGATACAACACA
	VvPAO2	VIT_206s0004g00610.1	F:AGAGTCACAGCCAGGAAGAGAAG
			R:CACCACACCACCACATCAAGAC
	VvPAO3	VIT_206s0004g00620.1	F:CAAGAGAAAGCCGCATTCCAAGA
			R:GAGCCAACCTATGAGCACAAGC
	VvPAO4	VIT_206s0061g00790.1	F:TGAAGTTGTTGCGGTTGATGATGA
			R:GCGATTAGTGTCCAGGTCCTCA
	VvPPH1	VIT_204s0023g02010.1	F:GCTCTTCTTCAATCACAACCGACT
			R:ACCCGCAGCAACGATTCCTT
	VvPPH2	VIT_213s0158g00180.2	F:ATCCTGCTCCTCAATCCAAGAA
			R:GGTCCTGCCATAAGTCAATAGAG
	VvPPH3	VIT_216s0022g01340.1	F:AGAGAGGAGCCCAGAGGTTGT
			R:GGTGAGGTTATAGGCAGTTGAAGC

	VvFtsH6-1	VIT_212s0028g01600.1	F:GGTGAGCCTGAGGTGACAACA
			R:TGAGCCGAGGTGTCCATAAGC
	VvFtsH6-2	VIT_214s0108g00590.1	F:CCAGAGAGCCCAGTTGAATCCA
			R:ATCTCGGCAATCGCAACAGTTC
ABFs	*VvZIP08*	VIT_203s0063g00310.1	F:GGGCTACAGAGTGGAGGAATGG
			R:GAGCAGGCGATAATGAAGATGTGT
	VvZIP25	VIT_208s0007g03420.1	F:ACCGTAAGTCCAGTGTCTTCCG
			R:TGTCTCCGTTCCACCACCTTC
	VvZIP45	VIT_218s0001g10450.1	F:GCAGAAAAATCAGGTTATGGAG
			R:AGCACAGCAAACAATTCCCT
ABA recptors	*VvRCAR1*	VIT_215s0046g01050.1	F:TGATGGGAGACCAGGGACAC
			R:TTTGAGGTTGCAGTTGATGAG
	VvRCAR2	VIT_216s0050g02620.2	F:GGATGTGAAAGTGGGAATGG
			R:GGAAGATGGAAGAAGGGCTAC
	VvRCAR3	VIT_202s0025g01340.2	F:GGCAAAGCATTTGAGGAAC
			R:GGGTAGCATTGAAAGGAAGAG
	VvRCAR4	GSVIVT00035869001	F:CTGTAATTGGAGGGGACCAC
			R:CACCACGTACGACTCCATCA
	VvRCAR5	GSVIVT00037390001	F:GTTTTTGTCGACACCATCGTT
			R:TCATGACGATGACCTCTTGC
	VvRCAR6	VIT_213s0067g01940.1	F:CGGGTAACACGAAGGAGGA
			R:GGGGTGCAATAATCTAAAAGAG
	VvRCAR7	VIT_202s0012g01270.1	F:ACTACCGGTCGGTGACTACG
			R:TCCTCTGTGTTCCCTTCTGG
PP2C	*VvPP2C4*	VIT_211s0016g03180.1	F:TGGGCTTTGGGATGTTATGT
			R:TGTGCAGGAGTCTCATCAGC
	VvPP2C9	VIT_206s0004g05460.2	F:TTAAAGCCCTTCGTGAGCTG
			R:GACACCACGTCCCACAGAC
SnRK	*VvSnRK2.1*	VIT_202s0236g00130.1	F:TTTTTGTGGCAAACCCAGAT
			R:CAGCTTCCTCCATCCATCAT
	VvSnRK2.3	VIT_212s0035g00310.1	F:AGCTCTTTGAGCGAATCTGC
			R:CATGGAATGGCAATAGCTGA
